# The Value of First-Order Features Based on the Apparent Diffusion Coefficient Map in Evaluating the Therapeutic Effect of Low-Intensity Pulsed Ultrasound for Acute Traumatic Brain Injury With a Rat Model

**DOI:** 10.3389/fncom.2022.923247

**Published:** 2022-06-23

**Authors:** Dan Du, Yajuan Gao, Tao Zheng, Linsha Yang, Zhanqiu Wang, Qinglei Shi, Shuo Wu, Xin Liang, Xinyu Yao, Jiabin Lu, Lanxiang Liu

**Affiliations:** ^1^Department of Magnetic Resonance Imaging, Qinhuangdao Municipal No. 1 Hospital, Qinhuangdao, China; ^2^Department of Radiology, Peking University Third Hospital, Beijing, China; ^3^NMPA Key Laboratory for Evaluation of Medical Imaging Equipment and Technique, Beijing, China; ^4^Peking University Shenzhen Graduate School, Shenzhen, China; ^5^MR Scientific Marketing, Siemens Healthineers Ltd., Beijing, China; ^6^Graduate School of Chengde Medical University, Chengde, China; ^7^Beijing Key Laboratory of Magnetic Resonance Imaging Device and Technique, Beijing, China

**Keywords:** low-intensity pulsed ultrasound, acute traumatic brain injury, ADC map, neuroprotective, first-order feature

## Abstract

**Purpose:**

In order to evaluate the neuroprotective effect of low-intensity pulsed ultrasound (LIPUS) for acute traumatic brain injury (TBI), we studied the potential of apparent diffusion coefficient (ADC) values and ADC-derived first-order features regarding this problem.

**Methods:**

Forty-five male Sprague Dawley rats (sham group: 15, TBI group: 15, LIPUS treated: 15) were enrolled and underwent magnetic resonance imaging. Scanning layers were acquired using a multi-shot readout segmentation of long variable echo trains (RESOLVE) to decrease distortion. The ultrasound transducer was applied to the designated region in the injured cortical areas using a conical collimator and was filled with an ultrasound coupling gel. Regions of interest were manually delineated in the center of the damaged cortex on the diffusion weighted images (*b* = 800 s/mm^2^) layer by layer for the TBI and LIPUS treated groups using the open-source software ITK-SNAP. Before analysis and modeling, the features were normalized using a z-score method, and a logistic regression model with a backward filtering method was employed to perform the modeling. The entire process was completed using the R language.

**Results:**

During the observation time, the ADC values ipsilateral to the trauma in the TBI and LIPUS groups increased rapidly up to 24 h. After statistical analysis, the 10th percentile, 90th percentile, mean, skewness, and uniformity demonstrated a significant difference among three groups. The receiver operating characteristic curve (ROC) analysis shows that the combined LR model exhibited the highest area under the curve value (AUC: 0.96).

**Conclusion:**

The combined LR model of first-order features based on the ADC map can acquire a higher diagnostic performance than each feature only in evaluating the neuroprotective effect of LIPUS for TBI. Models based on first-order features may have potential value in predicting the therapeutic effect of LIPUS in clinical practice in the future.

## Introduction

Traumatic brain injury (TBI) is fatal and disabling not only due to direct trauma, but also progressive secondary injuries, such as cytotoxic edema, vasogenic edema, microstructural changes, and diffuse axonal injury (Werner and Engelhard, 2007; Blennow et al., 2012), which can exacerbate the extent of brain trauma and affect prognosis. It is particularly important to control and ameliorate these secondary injuries to improve the diagnosis, accuracy of injury staging, and evaluation of therapeutic approaches.

Diffusion-weighted magnetic resonance imaging (DWI) is based on the random and irregular Brownian motion of water molecules and reflects the different states of water molecules in tissues (Fernández-Espejo et al., 2011). It effectively reflects the microstructure and micromotion *in vivo* and provides the functional state of human tissues at the molecular level (Chavhan et al., 2014). Studies have shown that it is the only non-invasive method to detect the diffusion of water molecules in living tissues and has a high sensitivity, which can provide valuable information for the diagnosis of diseases (Koutoulidis et al., 2016). The magnitude of diffusion is indicated by the apparent diffusion coefficient (ADC), and a low ADC value indicates restricted diffusion, which is considered as high signal intensity on DWI. In addition, the presence of vascular edema, hemorrhage, and cystic changes leads to heterogeneity of ADC values (Takaya et al., 2021).

Low-intensity transcranial ultrasound stimulation (LIPUS) is a novel treatment for encephalopathy that modulates a variety of brain functions, such as protein expression, intracellular clearance of harmful substances, neuro-electrical signaling, and behavior, by a mechanism based on ultrasound-induced complex electromechanical interactions that induce current effects through changes in the membrane capacitance of neurons, leading to excitation (Plaksin et al., 2013). LIPUS has been shown to protect against brain damage and suppress chemically induced acute epileptic electroencephalogram (EEG) activity in a rat model of Alzheimer’s disease (Lin et al., 2015). In addition, it can alter Blood Oxygen Level-Dependent (BOLD) signals and specifically modulate brain activity in functional magnetic resonance imaging (fMRI)-monitored regions (Yoo et al., 2011). It can increase cerebral blood flow and protect the brain from ischemic injury (Xu et al., 2011; Guo et al., 2015). Other studies have attempted to detect extracellular parameters of TBI brain tissue by Gd-DTPA tracer technique and found that LIPUS treatment can significantly enlarge the extracellular gap of brain and improve the drainage of interstitial fluid in brain tissue, thus improving neuronal and glial cell edema and promoting the process of brain recovery after TBI (Li et al., 2013; Su et al., 2017). Yuan’s study demonstrated that LIPUS can reduce ADC values, alter the diffusion of water molecules and thus neuromodulation (Yi et al., 2017).

For many years, MRI-based computer-aided diagnosis has been shown to help with early screening and prediction of cognitive decline (Wong et al., 2021). Histogram analysis based on pixel distribution as a new parametric map analysis technique, mainly applied to tumors, can provide quantitative information on tumor heterogeneity, which is advantageous in the identification and grading of tumors in different organs and predicting treatment response (Woo et al., 2014; Yan et al., 2018). It has also been used by researchers in cerebral leukomalacia and cerebral ischemia (Bence et al., 2014; Bin et al., 2017). ADC histogram analysis can provide many parameters reflecting tissue characteristics such as hypoxia, angiogenesis, and cell proliferation in tumor lesions (Cho et al., 2015), or edema and neovascularization in inflammatory diseases (Wu et al., 2015; Makanyanga et al., 2017); however, there are no studies using histogram analysis of ADC maps to assess brain tissue after brain trauma and the local microscopic changes in microstructure and environment. Thus, our study assessed the effectiveness of LIPUS in recovering from traumatic brain injury by quantitatively measuring and analyzing ADC values and ADC map histogram parameters based on the volume at the trauma in the murine brain, in combination with immunohistochemistry.

## Materials and Methods

### Animals and Experimental Groups

In this study, 45 male Sprague Dawley (SD) rats (average weight: 250 g; average age: 2 months) were included. The animals were maintained at a temperature of 20–22^°^C and 60% air humidity, with *ad libitum* access to food and water. All rats were intraperitoneally injected with pentobarbital sodium (3%, 5 mg/100 g) before the operation. These experiments and conditions were in accordance with international ethical regulations and laws for the protection of animals and were approved by the Medical Ethics Committee and the Animal Care of Qinhuangdao Municipal No. 1 Hospital in China (No. 20140018).

Traumatic brain injury models were developed by performing a craniocerebral injury (CCI) operation. The rats were randomly divided into three groups (15 rats/group): the TBI group, LIPUS treatment group, and sham operation control group (sham group). Rats in the TBI group underwent only the CCI operation, and those in the sham group underwent scalp incision and skull drilling without CCI operation. Rats in the LIPUS group underwent CCI surgery followed by LIPUS treatment.

### MR Protocol

The rats were scanned using a 3.0-T MRI system (MAGNETOM Verio, Siemens Medical Solutions, Erlangen, Germany) at 3 h, 24 h, 48 h, 72 h and 7 d after trauma. Specific animal MRI coils (4 channel high resolution, diameter 50 mm, P/N 10-F04885, Shenzhen RF Tech Co., Ltd.) were used in this study. All rats were intraperitoneally injected with pentobarbital sodium (3%, 5 mg/100 g, IP) before operation. The scanning layers were aligned parallel to the anterior/posterior line and acquired using a multi-shot readout segmentation of long variable echo trains (RESOLVE) to decrease the distortion. The MR parameters were as follows:

T2-weighted imaging (T2WI): repetition number (TR) = 4,000 ms, echo time (TE) = 113 ms, average = 6, layer thickness = 2.0 mm, layer number = 10, voxel size = 0.3 × 0.3 × 2.0 mm, field of view (FOV) = 65 × 65 mm, and flip angle = 150^°^.

RESOLVE-DWI (RS-DWI): repetition time (TR) = 5,000 ms; echo time (TE) = 70 ms, layer thickness = 2.2 mm, layer number = 15, voxel size: 1.0 mm × 1.0 mm × 2.2 mm, field-of-view [FOV]: 98 mm × 98 mm, flip angle = 150^°^, and *b*-values: 0 and 1,000 s/mm^2^.

Imaging analysis was carried out using prototype software on a workstation (Siemens Verio 3.0T MR Leonardo 3682). The ADC map images of mouse brains were co-registered with T2WI for exactly identification. ADC values of the injured area and the contralateral side were measured by freehand region of interest method, at different time periods.

### Low-Intensity Pulsed Ultrasound Protocol

In the LIPUS system, two connected function generators (AFG3022C; Tektronix, United States) were used to generate the pulsed signals. The pulsed signal from the second generator was amplified using a linear power amplifier (E&I240 L; ENI Inc., United States) and transmitted to an unfocused ultrasound transducer (V301-SU; Olympus, Japan). Rats were anesthetized with an intraperitoneal injection of sodium pentobarbital (3%, 5 mg/100 g, IP). The ultrasound transducer was applied to the designated region in the injured cortical areas using a conical collimator with a diameter of 10 mm and was filled with ultrasound coupling gel. The total stimulation duration was 10 min. LIPUS was administered immediately to the rats and the rats were treated with LIPUS stimulation once per day for 7 d. The ultrasound fundamental frequency (FF) and pulsed repetition frequency (PRF) were 500 and 1 kHz, respectively. The ultrasound stimulation duration (SD) and tone-burst duration (TBD) were 400 and 0.5 ms, respectively (Li et al., 2017). The ultrasound pressure was measured using a calibrated needle-type hydrophone (HNR500; Onda, United States), and the spatial peak and pulse-average intensity (Isppa) was 2.6 W/cm2. The method is similar to our previous study (Wu et al., 2020).

### Image Analysis

**FIGURE 1:**
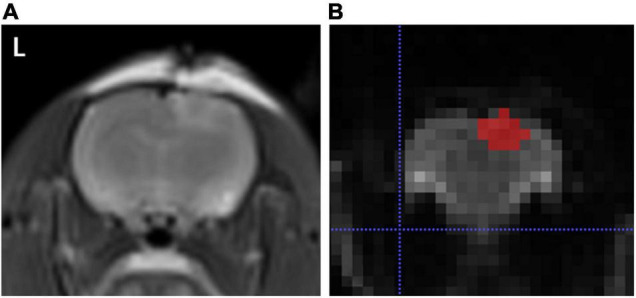
**(A)** Represents T2WI sequence, and **(B)** indicates a representative layer of brain trauma areas outlined by the application software ITK-SNAP.

After digital transfer of the ADC map data from the picture archiving and communication system (PACS) workstation to a personal computer, analysis was performed using ImageJ^1^ and a software application named ITK-SNAP. The features were extracted using an open source tool named Pyradiomics^2^ with the following sequences: normalize: true, normalize Scale: 100, interpolator: sitkB spline, resampled pixel spacing: [2 2 2], bin Width: 25, voxel Array Shift: 30, correct Mask: True. Regions of interest (ROIs) were drawn in each slice of the ADC map by one author along the margin of the injury, including all slices in which the injury was visualized (Figure 1). Damage boundaries were identified with reference to the location and extensions of T2W images.

Apparent diffusion coefficient histogram processing process: (1) z-score normalization of the data; (2) LR modeling using backward rejection; (3) statistical analysis of the diagnostic value of each eigenvalue filtered and the total model and plotting the nomogram and ROC curves of the synthetic model. The entire process was completed using the R language.

### GFAP Staining

Fifteen rats were randomly selected per group, referring to TBI and LIPUS group, which with three rats per time point, respectively (*n* = 3, per time point, i.e., 3 h, 24 h, 48 h, 72 h, 7 d post-injury), and sham group with one rat per time point (*n* = 5). After MR scanning at each time point (3 h, 24 h, 48 h, 72 h, and 7 d), the rats were euthanized under ether anesthesia, and their brains were collected for immunohistochemical analysis.

Brain tissue was fixed in 10% formalin buffer, paraffin-embedded, and cut into 4 μm-thick sections. After dewaxing, sections were treated with 3% hydrogen peroxide (H_2_O_2_) in methanol at 40^°^C for 5–10 min. Sections were thoroughly washed and closed with 5% normal goat serum (NGS) for 2 h. Sections were rinsed in PBS and incubated with horseradish peroxidase (HRP) 1:500 dilution of primary antibody (rabbit-anti-cow GFAP) overnight at 4^°^C. Sections were then treated with enzyme-labeled anti-rabbit IgG (1:100) for 2 h at room temperature, followed by reaction with 3,3′-diaminobenzidine (DAB) (Sigma). DAB was used as a chromogenic agent, and hematoxylin was prepared and stained for another 3 min (Borges et al., 2003).

The extent of gliosis was scored semi-quantitatively for each brain by a blinded investigator assessing GFAP staining intensity on a 0–3 scale. A score of 0 was given to the regions that appeared normal with barely detectable staining, and score of 1, 2, and 3 were assigned to areas with little, moderate, and strong intensity GFAP immunoreactivity, respectively.

Four randomly selected sections of each section were observed under a 400× light microscope, and DFAP staining was scored semi-quantitatively and compared between groups. After immunohistochemical staining, the tissue sections were observed under a microscope by two experienced observers who had no knowledge of the experimental conditions.

### Statistical Analysis

Statistical analysis was conducted using the standard software package (GraphPad Prism v.7.00, La Jolla, CA, United States). A two-tailed *P* value of <0.05, was considered to indicate a statistically significant difference. ADC values were compared using repeated-measures analysis of variance, followed by Tukey’s test. One-way analysis of variance with Tukey-Kramer *post hoc* comparisons was used to reflect the situation of the TBI and LIPUS groups in the histogram parameters. The unpaired Student’s *t*-test was used to compare the histogram parameters between TBI and LIPUS group. Receiver operating characteristic (ROC) curve analysis was performed to analyze the diagnostic value of each important parameter for LIPUS treatment of traumatic brain injury.

## Results

### MRI Examination After Brain Trauma

**FIGURE 2:**
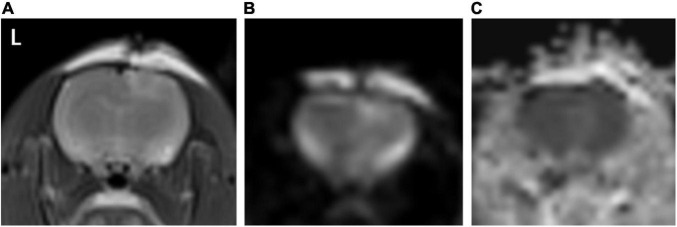
**(A–C)** Represents T2WI, DWI, and ADC map, respectively. As seen in image **(A,B)** a slightly high signal is seen in the T2WI and DWI sequence in the right cortex of the rat brain (ipsilateral to the trauma), and the same position in image **(C)**, no clear signal elevation or decrease, is seen.

T2WI and DWI sequences showed a slightly higher signal in the brain parenchyma ipsilateral to the trauma and no signal abnormality on the contralateral side, while ADC maps showed no clear signal abnormality both ipsilateral and contralateral to the trauma (Figure 2).

### Apparent Diffusion Coefficient Values of Injury Cortex

**FIGURE 3:**
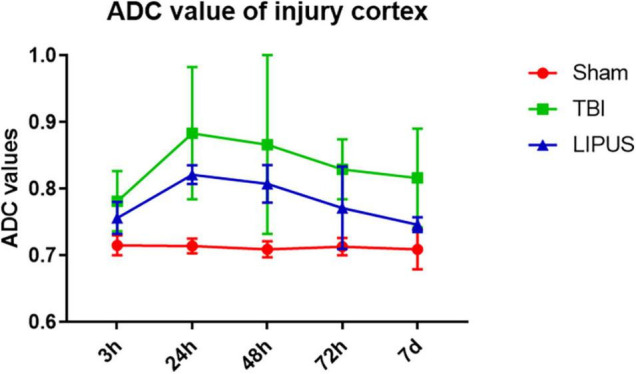
Variation trends of ADC values ipsilateral to the trauma in the TBI, LIPUS, and sham groups. Two-way ANOVA for repeated measurements, followed by Tukey’s *post hoc* test.

During the observation period, the ADC values ipsilateral to the trauma in the TBI and LIPUS groups increased rapidly up to 24 h and decreased slowly thereafter, while the sham group remained flat (Figure 3).

Apparent diffusion coefficient values on the trauma side were higher in both the TBI and LIPUS groups at 3 h compared to the sham group (0.781 ± 0.045 vs. 0.756 ± 0.024 vs. 0.715 ± 0.015), with a significant difference between the TBI and sham groups (*adjusted P value* = 0.0030) and no significant difference (*adjusted P value* = 0.0998).

With increasing time, ADC values peaked at 24 h in both the TBI and LIPUS groups, with significant differences between all three groups (Sham vs. TBI *adjusted P value* < 0.0001; Sham vs. LIPUS *adjusted P value* < 0.0001; TBI vs. LIPUS *adjusted P value* = 0.0058), but the LIPUS group peaked lower than the TBI group (0.821 ± 0.014 vs. 0.883 ± 0.099).

**FIGURE 4:**
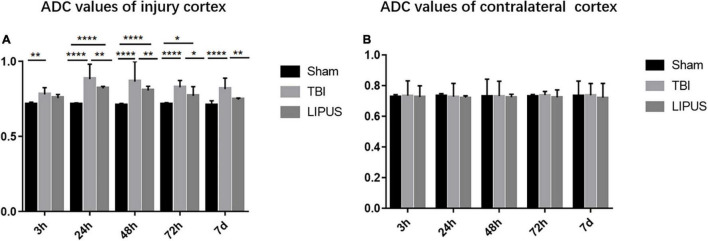
Changes in ipsilateral and contralateral ADC values of trauma at various time points in the Sham, TBI, and LIPUS groups, and there is no significant difference in contralateral cortex. Two-way ANOVA for repeated measurements, followed by Tukey’s *post hoc* test. *P < 0.05, **P < 0.01, ***P < 0.001, and ****P < 0.0001.

This was followed by a slow decline in ADC values, which remained higher than that in the sham group at 7 d. However, at 7 d, ADC values were lower in the LIPUS group than in the TBI group, closer to the sham group, and significantly different from the TBI group (*adjusted P value* = 0.0015), as shown in Figure 4.

### The First-Order Features Based on Apparent Diffusion Coefficient Maps

After statistical analysis, the 10th percentile, 90th percentile, mean, skewness, and uniformity demonstrated significant differences among the three groups (Table 1). The 10th percentile, 90th percentile, mean, and skewness were higher in the TBI group than in the LIPUS group (*P* = 0.024, 0.001, 0.002, and 0.035, respectively), while the mean value of uniformity was lower than that of the LIPUS group (*P* = 0.002).

**TABLE 1:** Compared 10 percentile, 90 percentile, mean, skewness, and uniformity between TBI group and LIPUS group (*P* < 0.05).

Parameters	Groups	Mean	SD	*P*-values
10 Percentile	TBI	741.65	209.49	0.024
	LIPUS	545.70	206.37	
90 Percentile	TBI	1275.24	256.46	0.001
	LIPUS	978.94	152.82	
Mean	TBI	1002.32	189.69	0.002
	LIPUS	756.77	174.32	
Skewness	TBI	0.41	0.62	0.035
	LIPUS	0.07	0.47	
Uniformity	TBI	0.02	0.00	0.002
	LIPUS	0.03	0.01	

### The Combined LR Model and Concerned Features

**FIGURE 5:**
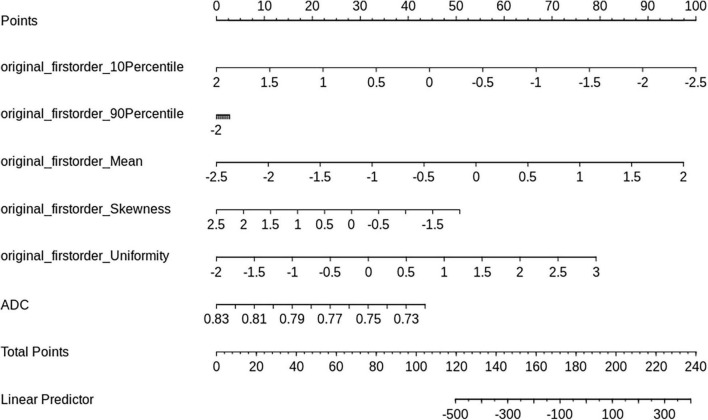
Nomogram based on the LR model.

**FIGURE 6:**
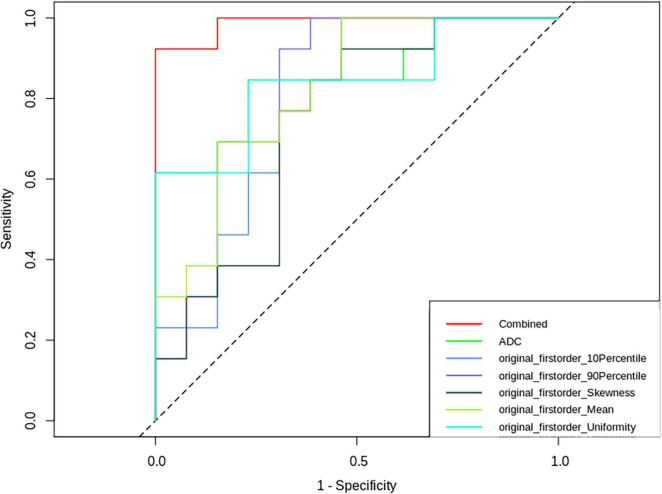
Receiver operating characteristic (ROC) curves of ADC-derived first-order features, ADC values and combined parameters.

The nomogram (Figure 5) and ROC analysis (Figure 6) showed that the combined LR model exhibited the highest area under the curve (AUC) value with the largest area under the ROC curve (AUC: 0.96) (Table 2). The AUCs of other features were lower than those of the combined model in descending order: ADC-AUC (0.7) > uniformity-AUC (0.68) > 90th percentile-AUC (0.65) = mean-AUC (0.65) > 10th percentile-AUC (0.59) > skewness-AUC (0.53).

**TABLE 2:** Efficacy of histogram parameters, ADC values, and the combination of the two in assessing the effectiveness of LIPUS treatment.

Parameters	AUC	CI	Sensitivity	Specificity
10 Percentile	0.59	(0.78, 0.96)	1	0.53
90 Percentile	0.65	(0.82, 0.99)	0.92	0.69
Mean	0.65	(0.82, 0.98)	0.69	0.84
Skewness	0.53	(0.73, 0.94)	0.84	0.61
Uniformity	0.68	(0.84, 1)	0.84	0.76
ADC	0.7	(0.85, 1)	0.84	0.76
Combined	0.96	(0.98, 1)	0.92	1

### GFAP Staining

**FIGURE 7:**
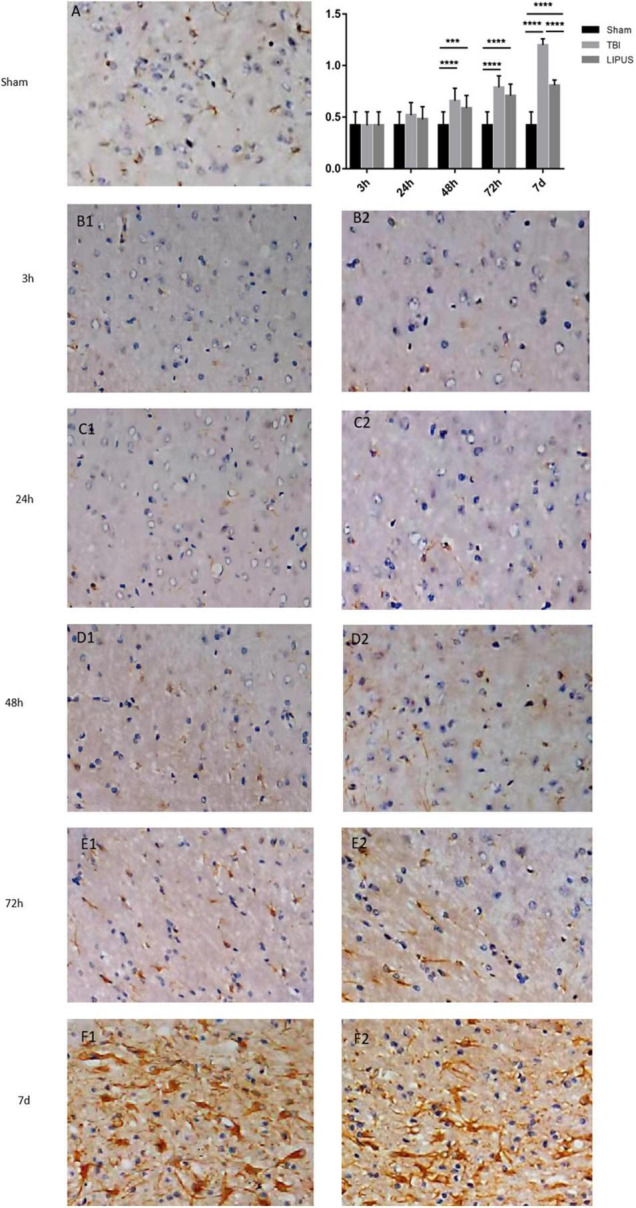
GFAP staining of rat brain cortex in the TBI **(B1–F1)**, LIPUS **(B2–F2)** and sham **(A)** groups at each time point. Representative images of GFAP-stained neurons in the injured lateral cortex. Compared with rats in the sham group, the number of GFAP-positive cells in the injured cortex was significantly increased in the TBI group and the number of positive cells expressed in the LIPUS group was significantly less than that in the TBI group, indicating that LIPUS has a good inhibitory effect on the growth of astrocytes and the formation of reactive gliosis. Data are Mean ± SD, *n* = 15. Unpaired *t*-test, ***P < 0.001, ****P < 0.0001.

GFAP staining showed that the neuronal staining in the sham group showed clear cytoplasmic nuclei, while the TBI and LIPUS groups showed swollen cells with few cells and sparse arrangement, and there were few GFAP-positive cells at 3 h. With the extension of time, most GFAP-positive cells were expressed at 7 d, but the LIPUS group showed weaker expression of GFAP positive cells than the TBI group (Figure 7). The results of semi-quantitative analysis showed that the GFAP staining score was 0.42 ± 0.13 (mean ± SD) in the sham group and gradually increased with time in the TBI and LIPUS groups until 7 d when the score was highest (1.20 ± 0.06 vs. 0.81 ± 0.05) and statistically different (TBI vs. LIPUS *adjusted P <* 0.0001).

## Discussion

MR-DWI plays an important role in the diagnosis and treatment of central nervous system disorders such as acute cerebral ischemia, brain tumors, brain abscess staging, and diffuse axonal injury, especially in quantitative analysis using ADC values (Schaefer et al., 2000). Hou et al. (2007) and Long et al. (2015) used the ADC values measured by DWI images to confirm that DWI has great advantages in studying brain edema due to TBI. The ADC values can quantify the recovery before and after treatment of brain injury, and the higher the degree of injury, the greater the ADC values (Hou et al., 2007; Long et al., 2015). In particular, for visible lesions that are not clearly defined on anatomical scans (Braeckman et al., 2019), quantitative measurements can reflect the microscopic conditions of brain tissue. Assaf et al. (1997) found a decrease in ADC values 1 h after injury in a closed craniocerebral injury model, which may be related to the formation of early cytotoxic edema. Barzo et al. observed a 1 h post-traumatic brain water content and transient increase in ADC (Donald et al., 2007). Hanstock et al. (1994) showed an acute phase elevation in ADC values due to cytotoxic edema and selective results of vasogenic edema.

### Diagnostic Performance of Apparent Diffusion Coefficient Values

Our findings revealed that ADC values in the traumatized lateral cortex of rats in the TBI group increased at 3 h after brain trauma, peaked at 24 h, and then gradually decreased. Analysis of the early ADC value elevation was attributed to disruption of the blood-brain barrier, causing vasogenic edema (Baldwin et al., 1996). Our results were also confirmed by the findings of Immonen et al. (2009) that cytotoxic edema began to subside at 3 h post-injury. This is similar to the results of Long et al. (2015), who found an increase in ADC values at 3 h. However, our results were in contrast to those of Benedict et al., who found that 3 and 24 h post-trauma cortical ADC values were the same as pre-trauma and then gradually increased (Albensi et al., 2000). The reason for this may be due to the different models chosen; Benedict et al. applied the fluid percussion model, while our study applied the controlled cortical impact (CCI) model.

Apparent diffusion coefficient values in the LIPUS group also peaked at 24 h, but the peak was lower than that of the TBI group, and the time profile after 24 h also showed a slow decline, with ADC values converging to those of the sham group at 7 d. The diffusive movement of water molecules *in vivo* includes extracellular, intracellular, and intercellular movements, as well as microcirculation. The main reason for the changes in ADC values is due to alterations in extracellular movement and microcirculation. Ultrasound reduces the random movement of water molecules (Tufail et al., 2010), causes neuronal excitation, leads to astrocyte swelling, and is accompanied by up to 30% reduction in the extracellular gap (Shi et al., 2015). The reduction in extracellular gaps would limit the diffusion of molecular water; thus, in comparison with the TBI group, LIPUS was found to be able to reduce ADC values, and at 7 d, the ADC values of the LIPUS group were close to those of the sham group, further confirming the ability of LPIUS to play a cerebral protective role.

Because traumatic brain injury is a heterogeneous injury and the mechanism of injury determines its complexity, conventional ADC values do not reflect the heterogeneity of the post-traumatic brain injury, and the selection of a region in a representative traumatic brain tomographic image for measurement and analysis is prone to sampling bias (Bharwani et al., 2011), which may be the reason for the difference in elevated or reduced ADC values. Therefore, whole brain trauma volume analysis is more representative of posttraumatic heterogeneity than the maximum cross-sectional area and can eliminate sampling bias to a large extent.

### Diagnostic Performance of First-Order Features Based on Apparent Diffusion Coefficient Maps

First-order statistics describe the distribution of voxel intensities. Our study found that the 10th percentile, 90th percentile, mean, and skewness were higher in the TBI group than in the LIPUS group, and that the 10th percentile, 90th percentile, mean, and ADC values varied similarly and will not be described further. Skewness, which is a measure of the asymmetry of the histogram, is positive if the majority of the data are concentrated on the left of the histogram and negative if the majority of data is concentrated on the right. Skewness responds to the symmetry of the data relative to the mean and can be positive or negative depending on the trend of concentration in the histogram and the direction of the “delay” in the tail of the histogram. This indicator describes the shape of the histogram. Generally, the more complex the structure of the organization, the more the signal strength is distributed in the tails and the higher is the skewness value. Uniformity is a measure of the sum of squares of each intensity value. This is a measure of the homogeneity of the image array, where a greater uniformity implies greater homogeneity or a smaller range of discrete intensity values. Uniformity is the sum of the squares of the signal intensities of all pixels in the region of interest. The closer the signal intensities of the pixels in the region of interest, the smaller the uniformity. Ionic, molecular, and cellular changes are triggered immediately after brain trauma, with a large increase in extracellular water content, progressive loss of proteins from the extracellular matrix, increased amount of cellular degeneration, and increased mobility of water molecules (Immonen et al., 2009), resulting in a complex tissue structure, an increased skewness value, and a smaller uniformity for brain cells that are mostly in a damaged state. Early application of LIPUS is effective in promoting blood-brain-barrier recovery and increasing water transport in the blood-brain barrier (Yoon et al., 2012), which will help reduce brain edema, and reduce neutrophil aggregation, thereby reducing the release of inflammatory mediators, protein hydrolases, and reactive oxygen species and reducing edema (Chen et al., 2018). LIPUS has also been shown to inhibit microglial activation, suppress a range of inflammatory immune responses, reduce cell swelling in brain tissue after injury, decrease cell membrane permeability, increase axon density, and decrease the tissue component of the extracellular space (Sato et al., 2015). This shows that LIPUS can reduce the heterogeneity of the extracellular space, with less brain cell damage and less uniformity, which explains the lower skewness values and higher uniformity values in the LIPUS group than in the TBI group.

### Diagnostic Performance of the Combined LR Model and Concerned Features

A nomogram can provide quantitative prognostic assessment in a dynamic manner. Currently, nomogram clinical prognostic prediction methods have been applied to a variety of diseases, such as oncology and other diseases (Liang et al., 2015; Devin et al., 2018; Tunthanathip et al., 2019). The model is a mathematical equation that connects predictors and outcomes of interest using a two-dimensional graphical scale. In our study, the nomogram was applied to score each indicator and thus predict the efficacy of LIPUS treatment after traumatic brain injury.

Radiomics is a new technology that automatically extracts large amounts of representative image data through algorithms and converts them into a feature space that can be utilized to reflect the microscopic characteristics of tumors (Gillies et al., 2016), and has played an important role in the identification and clinical application of imaging biomarkers (Makanyanga et al., 2017). In this study, we found that among the statistically significant first-order parameters, although the sensitivity of the 10th percentile was 100%, the specificity was low at 53%. The uniformity AUC value was higher at 0.68, and its sensitivity and specificity were on par with the ADC values at 84 and 76%, respectively. The combined LR model had the largest area under the ROC curve at 0.96, and its sensitivity and specificity were 92 and 100%, respectively.

### GFAP Staining of Injury Cortex in the Traumatic Brain Injury, Low-Intensity Pulsed Ultrasound and Sham Group

GFAP revealed a significant increase in the number of damaged cortical GFAP-stained cells in the TBI group and significantly less cell damage in the LIPUS group than in the TBI group, but more than that in the sham group. As mentioned earlier, the immediate destruction of brain tissue after traumatic brain injury triggers a number of molecular and biochemical pathways, including astrocyte proliferation and inflammation (Greve and Zink, 2009; Karve et al., 2016). GFAP, a component of mature astrocyte intermediate filaments, not only plays a role in regulating cellular metabolism, forming and maintaining the blood-brain barrier, and producing and releasing neurotrophic factors (Zoltewicz et al., 2012).

Injury-induced astrocyte proliferation often includes overexpression of GFAP, which is used as a biomarker to study injury progression (Pekny and Pekna, 2014). TBI causes damage to astrocytes, which in turn induces GFAP expression and shows variability depending on the duration and extent of injury (Boyen and Gb, 2004; Zhang et al., 2012). As astrocytes die, polymers of GFAP break down (Middeldorp and Hol, 2011), and astrocytes rapidly divide, proliferate, and repair in a glial scar-forming manner. The dense barrier formed by the proliferating scar hinders the regeneration and functional reconstruction of nerve tissue and is one of the important factors affecting nerve regeneration and functional reconstruction. Studies have been conducted at home and abroad using animal experiments to inhibit astrocyte growth and formation of reactive gliosis by inhibiting GFAP gene expression, thereby promoting functional reconstruction of nerve regeneration (Hausmann et al., 2000). Susarla et al. (2014) found that the value of GFAP-positive astrocytes peaked in the acute phase. Our study compared GFAP staining in the TBI, LIPUS, and sham groups and found that on day 7 post-trauma, there was a statistical difference in the number of GFAP-stained cells between the groups, and the number of damaged cortical GFAP-stained cells increased significantly in the TBI group, and the number of damaged cells in the LIPUS group was significantly lower than in the TBI group but more than in the sham group, which is consistent with the Neha Soni study, which found that the positive expression of GFAP in the cortex on the traumatized side reached a high value (Soni et al., 2018) 7 days after trauma. This further demonstrates that LIPUS has a good inhibitory effect on astrocyte growth and reactive gliosis formation.

### Limitations

This study had a few limitations. First, the sample size was small; future studies with larger sample sizes should be conducted to determine the reproducibility of the results. Second, all ROIs were determined manually, and there were some challenges in determining murine brain trauma boundaries, which might have resulted in measurement errors.

## Conclusion

The application of LIPUS treatment in the acute phase can effectively alleviate brain edema caused by blood-brain barrier disruption, reduce inflammatory response, inhibit astrocyte growth and reactive gliosis, and provide a theoretical basis for brain trauma prognosis and brain rehabilitation. What’s more, the combined LR model of first-order features based on the ADC map can acquire a higher diagnostic performance than each feature only in evaluating the neuroprotective effect of LIPUS for TBI.

## Data Availability Statement

The raw data supporting the conclusions of this article will be made available by the authors, without undue reservation.

## Ethics Statement

The animal study was reviewed and approved by the Medical Ethics Committee and Animal Care of Qinhuangdao Municipal No. 1 Hospital in China (No. 20140018).

## Author Contributions

LL, ZW, and DD designed the study. DD, TZ, and SW performed the experiments. DD, YG, TZ, QS, LSY, SW, JL, XL, and XY analyzed the data. DD wrote the manuscript. All authors approved the final version of the manuscript.

## Conflict of Interest

QS was employed by Siemens Healthineers Ltd. The remaining authors declare that the research was conducted in the absence of any commercial or financial relationships that could be construed as a potential conflict of interest.

## Publisher’s Note

All claims expressed in this article are solely those of the authors and do not necessarily represent those of their affiliated organizations, or those of the publisher, the editors and the reviewers. Any product that may be evaluated in this article, or claim that may be made by its manufacturer, is not guaranteed or endorsed by the publisher.

## Footnotes

1
http://rsb.info.nih.gov/ij/

2
https://pyradiomics.readthedocs.io/en/latest/index.html
